# Cytotoxicity evaluation of different clear aligner systems on human primary gingival fibroblasts: an in vitro study

**DOI:** 10.2340/biid.v13.46006

**Published:** 2026-05-12

**Authors:** Rituparna Pronajit Das, Parag Vishnu Gangurde, Alok Ranjan, Shashank Sharad Gaikwad, Hitesh Ramdas Sawant, Manav Karia

**Affiliations:** a Bharati Vidyapeeth (Deemed to be University) Dental College and Hospital, Navi Mumbai, India; b Orthodontics and Dentofacial Orthopaedics, Bharati Vidyapeeth (Deemed to be University) Dental College and Hospital, Navi Mumbai, India

**Keywords:** clear aligners, cytotoxicity, MTT assay, biocompatibility, human gingival fibroblasts

## Abstract

**Background:**

Clear aligners, widely adopted in modern orthodontics, are designed to stay in constant contact with the oral mucosa for extended durations. Persistent concerns exist about the possible leaching of cytotoxic agents like bisphenol-A from these materials, which have heightened the need for rigorous biocompatibility evaluation. Among various assays, the MTT (3-(4,5-dimethylthiazol-2-yl)-2,5- diphenyltetrazolium bromide) assay is a reliable in vitro method, which assesses metabolic activity and cell viability, making it a gold standard for cytotoxicity screening.

**Objective:**

The objective was to assess and contrast the potential cell-damaging effects of three different clear aligner brands – Invisalign^®^, Smile^®^ Aligners, and Illusion^®^ Aligners using the MTT assay on human gingival fibroblasts (HGFs).

**Methods:**

Aligner pellets were immersed in normal saline for 30 days at 37°C. Eluents were diluted to 5, 10, and 20% concentrations. HGFs were exposed to these eluents for 48 hours, and cytotoxicity was assessed via MTT assay. To assess the survival rate of the cells, light absorbance was recorded at a wavelength of 570 nanometers.

**Results:**

Invisalign^®^ showed the highest biocompatibility (97–98% viability across concentrations), followed by Smile^®^ Aligners (94–99%). Illusion^®^ Aligners exhibited significant cytotoxicity (80–92% viability), with a dose-dependent decline (*p* < 0.05).

**Conclusion:**

Material composition critically influences cytotoxicity. Invisalign^®^ and Smile^®^ Aligners are a safe options, while Illusion^®^ Aligners require further refinement for clinical safety. These results highlight the need for transparency in material composition and standardization in manufacturing protocols for all aligner systems to ensure patient safety.

## Introduction

Clear aligner therapy (CAT) has revolutionized orthodontics by offering an esthetic alternative to fixed appliances. Since its introduction by Align Technology (San Jose, CA, USA) in the 1990s, CAT has become widely adopted due to advantages such as improved oral hygiene, reduced discomfort, and enhanced patient compliance [1, 2].

Currently, a variety of aligner systems are commercially available, employing different polymeric materials and manufacturing techniques. Thermoplastic polymers such as polyethylene terephthalate glycol (PETG), polyurethane, and polypropylene blends are commonly used due to their transparency and moldability. While manufacturers focus heavily on mechanical performance characteristics – such as force transmission, shape memory, and material resilience – the biological safety of these materials is an equally critical concern that often receives less attention [3, 4].

Aligners are worn intraorally for approximately 20–22 hours a day and often for several months to years. During this extended exposure, these materials are subjected to mechanical stress, salivary enzymes, pH fluctuations, and thermal changes. These factors can contribute to the leaching of residual monomers, plasticizers, stabilizers, and degradation by-products, which may induce local or systemic biological effects, including cytotoxicity and inflammation [3, 5]. Cytotoxic effects could impair gingival fibroblast function, leading to inflammation or tissue damage [6].

Human gingival fibroblasts (HGFs) are critical cells found in the connective tissue of the periodontium. They play key roles in maintaining gingival architecture, synthesizing extracellular matrix, and orchestrating wound healing and inflammatory responses. Because they are among the first cells to come in contact with leached substances from dental materials, HGFs are frequently used in in vitro cytotoxicity testing models [4, 7].

Cytotoxicity assays such as the MTT assay are widely used for their sensitivity to metabolic changes in cells exposed to leachables from thermoplastic materials [8]. The MTT (3-(4,5-dimethylthiazol-2-yl)-2,5-diphenyltetrazolium bromide) assay relies on the transformation of a yellow MTT compound into dark purple formazan crystals through the activity of living cells. The depth of the resulting color, measured using a spectrophotometer, reflects the amount of metabolically active cells present. It is a reliable, reproducible, and sensitive method for preliminary biocompatibility screening of dental biomaterials [9]. While genotoxicity and estrogenicity assays add depth, they are less suitable for routine screening due to the complexity of the procedure and the need for added resources. Cytotoxicity testing thus offers a practical bridge between material formulation and biological response [8].

Although a few previous studies have assessed the cytotoxicity of specific aligner systems, comparative data under standardized experimental conditions remain sparse [4, 7, 10]. There is an urgent need to investigate whether lower-cost or unregulated brands demonstrate biocompatibility levels comparable to established systems such as Invisalign^®^.

This study aims to fill this knowledge gap by comparing the cytotoxicity of three commercially available clear aligner systems – Invisalign^®^, Smile^®^ Aligners, and Illusion^®^ Aligners – on HGFs using the MTT assay. The study’s outcomes will help identify differences in biocompatibility between brands and raise awareness about the importance of material evaluation in orthodontics, particularly for long-term intraoral devices. The null hypothesis stated that no significant difference in cell viability would occur among the three aligner systems at varying eluate concentrations (5, 10, and 20%).

## Materials and methods

### Study design

This was a prospective, double-blind, in vitro experimental study designed to evaluate and compare the cytotoxic effects of three commercially available clear aligner systems – Invisalign^®^ (Align Technology, USA), Smile^®^ Aligners (Lingual Matrix Service Pvt Ltd, India), and Illusion^®^ Aligners (Laxmi Dental Ltd, India) on HGFs. The study employed the MTT assay as the primary test for cytotoxicity and was conducted at the Central Research Laboratory of Maratha Mandal’s Dental College and Research Centre (MMCRL), Karnataka, India.

This study was approved by the Institutional Ethics Committee, Bharati Vidyapeeth (Deemed to be University) Dental College and Hospital (Protocol Number BEC387082023 Version No. 001).

### Blinding

To ensure objectivity and minimize bias, a double-blind protocol was employed in this study. The aligner brands were concealed using sequentially numbered, opaque, sealed envelopes prepared and shuffled by an independent investigator not involved in testing. Both the primary investigator and the researcher performing the cytotoxicity assays were blinded to group allocation. Following completion of the assays, data were analyzed by a blinded statistician to ensure unbiased interpretation.

### Aligner sample preparation

**Figure 1:**
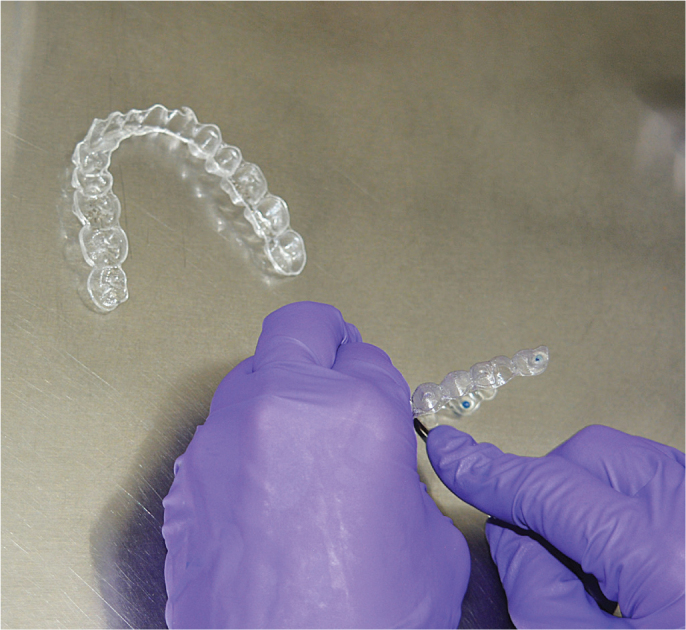
Samples being cut into pellets.

**Figure 2:**
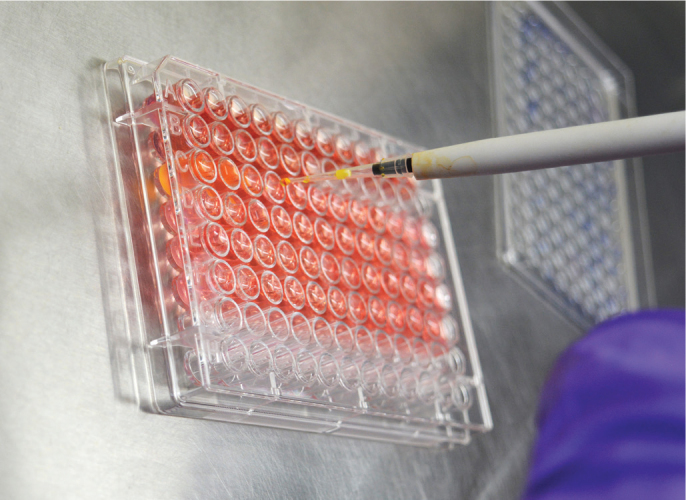
Introducing aligner serial immersion solutions (5, 10 and 20%).

One unused set of aligners (upper and lower tray) from each system was selected. Each set of aligners was sectioned into eight pellets (Figure 1). Each tray was sectioned at three standardized locations, between the central incisors and between the first and second premolars bilaterally to ensure uniformity across all aligner samples. The pellets were then placed in test tubes containing normal saline and incubated at 37°C for 1 month. The extracted solutions were adjusted to volume ratios of 5, 10, and 20% (Figure 2). A ceiling of 20% was set to avoid over-diluting the growth medium, which might disrupt normal cellular behavior [10]. These prepared concentrations were then applied to HGF cultures to observe any harmful cellular responses caused by the aligner materials.

### Gingival fibroblast cultures

For this investigation, gingival fibroblast cell lines (repository of MMCRL, Belgaum, Karnataka, India) were cultured in Dulbecco’s modified Eagle medium (DMEM; HiMedia Laboratories, Nashik, Maharashtra, India) supplemented with 10% heat inactivated fetal bovine serum (FBS; Gibco–BRL, Paisley, UK), non-essential amino acids, and an antibiotic–antimycotic solution (HiMedia Laboratories, Nashik, Maharashtra, India) containing 100 U/mL penicillin, 100 µg/mL streptomycin, and 0.25 µg/mL fungizone. The cells were seeded in flat-bottom 96-well culture plates at a density of approximately 1.5 × 10⁴ cells per well. The cell cultures were placed in an incubator set to 37°C with a humidified atmosphere containing 5% carbon dioxide and 85% moisture, allowing the cells to form a single, even layer within 48 hours. Before conducting any tests, the cultures were screened using a Polymerase Chain Reaction (PCR)-based detection method to confirm they were free of mycoplasma contamination. All experiments were performed using cells between passages P3 and P4 to ensure consistency.

### Cytotoxicity assessment using MTT assay

To assess cell toxicity, an MTT-based colorimetric method was employed, utilizing the conversion of a yellow tetrazolium compound into insoluble formazan by active cells. After removal of the culture supernatant, cells were exposed to eluents diluted to final concentrations of 5, 10, and 20% (v/v) in fresh DMEM. Untreated cells served as the control group treated with equivalent saline concentrations that had not been incubated with aligners. Following 48 hours of incubation under standard culture conditions (37°C, 5% CO₂), 20 µL of MTT stock solution (5 mg/mL in Phosphate Buffered Saline (PBS)) was added to each well and incubated for an additional 4 hours.

Subsequently, the supernatant was carefully aspirated, and the resulting formazan crystals were dissolved using 100 µL of dimethyl sulfoxide (DMSO). Optical density (OD) was measured at 570 nm using a microplate reader (LISA Plus) [11].

### Interpretation of results

Cell viability was calculated using the standard formula [12]:

Cell Viability (%) = (OD₅₇₀ e / OD₅₇₀ b) × 100

- OD₅₇₀ e represents the mean OD of the experimental (sample) group.
- OD₅₇₀ b represents the mean OD of the control group.

The reference group included cells grown solely in DMEM medium, without exposure to any aligner-derived substances. This group was used as the benchmark for full cell survival and as a comparison point for assessing potential toxic effects.

Cytotoxicity was graded based on the percentage of viable cells, following this scoring system [13]:

1. No cytotoxicity: >90% cell viability
2. Slight cytotoxicity: 60–90% cell viability
3. Moderate cytotoxicity: 30–59% cell viability
4. Severe cytotoxicity: <30% cell viability

### Statistical analysis

All statistical evaluations were conducted using SPSS software (version 26.0, IBM). To determine whether the numerical values followed a normal distribution, the Shapiro-Wilk test was applied, which indicated a deviation from normality. As a result, non-parametric methods were chosen for further analysis. The Kruskal-Wallis test was used to compare outcomes across the different aligner systems – Invisalign^®^, Smile^®^ Aligners, and Illusion^®^ Aligners – as well as the untreated control, at three concentration levels: 5, 10, and 20%. A single evaluator carried out all measurements, and each was repeated twice to enhance accuracy, with average values used for reporting. As OD measurements were obtained using an automated spectrophotometric method without subjective interpretation, formal intra-examiner reliability assessment was not required. A significance level of *p* < 0.05 was adopted, with an alpha error margin of 5% and a beta error of 20%, resulting in a study power of 80%.

## Results

The OD values and cell viability of clear aligners at different concentrations (5, 10, and 20%) revealed distinct patterns for the three aligner systems (Table 1).

**Table 1:** OD values and cell viability of clear aligners at different concentrations.

Aligner system	Concentration (%)	OD value (median)	IQR (Q1–Q3)	Cell viability (%)
Invisalign^®^	5	0.822	0.822–0.823	98.2
	10	0.819	0.818–0.821	97.9
	20	0.812	0.812–0.813	97.0
Smile^®^ Aligners	5	0.835	0.835–0.835	99.6
	10	0.798	0.796–0.799	95.2
	20	0.788	0.788–0.789	94.1
Illusion^®^ Aligners	5	0.767	0.766–0.768	91.5
	10	0.698	0.698–0.698	83.3
	20	0.668	0.667–0.669	79.7
Negative control	5	0.843	0.843–0.843	100
	10	0.839	0.838–0.839	100
	20	0.838	0.838–0.839	100

OD: optical density; IQR: interquartile range.

Invisalign^®^ showed highly consistent OD values at all concentrations (0.822, 0.819, and 0.812), with cell viability remaining above 97%. Statistical analysis confirmed that there was no significant decline, indicating excellent material stability and negligible cytotoxicity even at higher concentrations.

Smile^®^ Aligners demonstrated slightly lower OD values with increasing concentration (0.835 at 5%, 0.796 at 10%, and 0.788 at 20%), reflecting a mild but statistically significant dose-dependent reduction in cell viability (from 99 to 94%). Despite this trend, overall cytotoxicity remained within the slight cytotoxicity category.

In contrast, Illusion^®^ Aligners exhibited a pronounced concentration-dependent decline in OD (0.767 at 5%, 0.698 at 10%, and 0.668 at 20%), corresponding to a significant reduction in cell viability (from 92 to 80%). This finding indicates moderate cytotoxicity at higher concentrations and suggests the presence of leachable substances adversely affecting fibroblast survival.

The comparison between the groups using the Kruskal-Wallis test revealed a marked and statistically strong variation in the results, with the *p*-value falling below 0.01 for OD in A (Invisalign^®^), B (Smile^®^ Aligners), C (Illusion^®^ Aligners), and D (Negative Control) groups with a higher value in group D (Negative Control) (Table 2).

**Table 2:** Inter-group comparison of OD in A, B, C, and D groups.

Parameter	Groups	*N*	Median	Mean rank	Chi square value	*P* value of Kruskal-Wallis test
**OD**	A	24	0.820	52.50	61.28	0.001**
	B	24	0.800	44.50		
	C	24	0.700	12.50		
	D	8	0.840	76.50		

Kruskal-Wallis test;

** indicates statistically highly significant *p* < 0.01. OD: optical density. A: Invisalign^®^ Aligners; B: Smile^®^ Aligners; C: Illusion^®^ Aligners; D: Negative Control.

**Figure 3:**
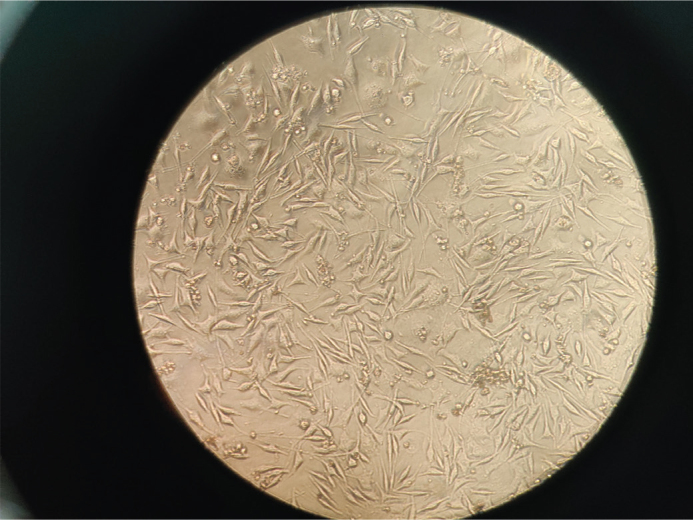
Microscopic picture of HGF cells treated with Sample A – Invisalign (20%). HGF: human gingival fibroblasts.

**Figure 4:**
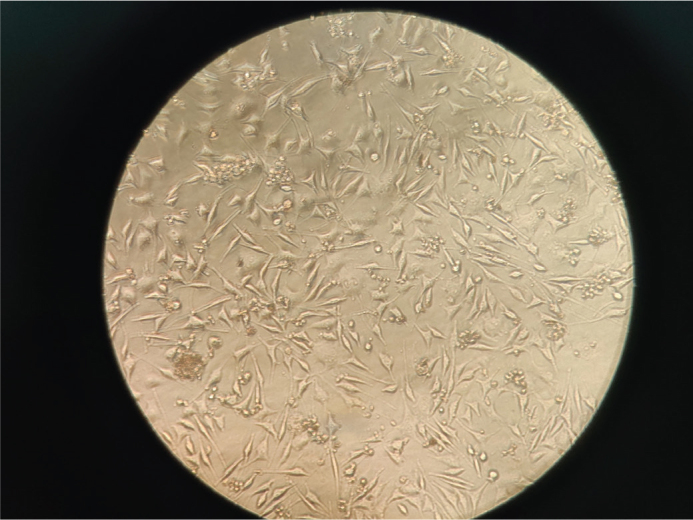
Microscopic picture of HGF cells treated with Sample B – Smile Aligners (20%). HGF: human gingival fibroblasts.

**Figure 5:**
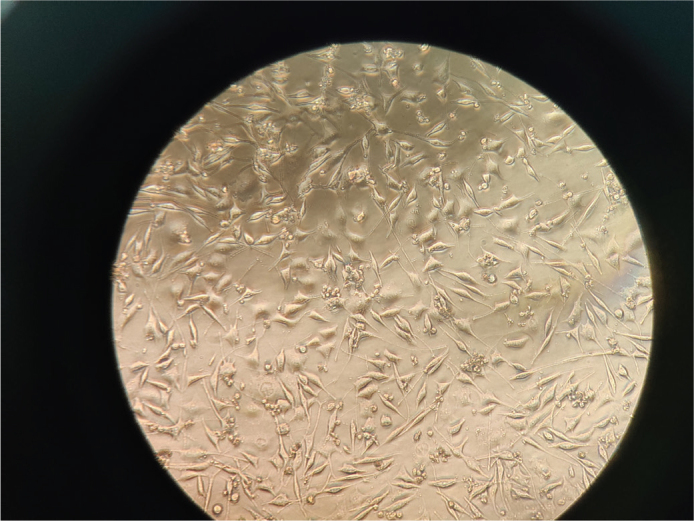
Microscopic picture of HGF cells treated with Sample C – Illusion Aligners (20%). HGF: human gingival fibroblasts.

**Graph 1:**
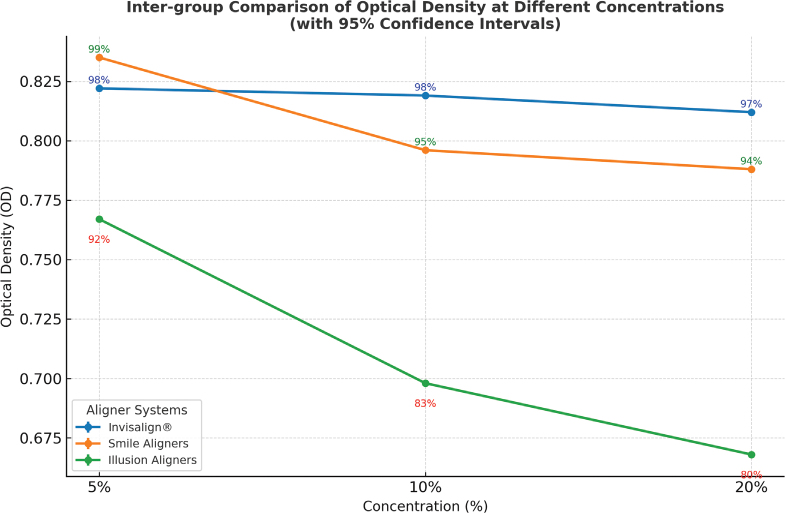
Inter-group comparison of OD at different concentrations (with 95% confidence intervals). OD: optical density.

Thus, the MTT assay results revealed significant differences in the cytotoxic profiles of the three tested clear aligner systems – Invisalign^®^, Smile^®^ Aligners, and Illusion^®^ Aligners – at all tested concentrations (5, 10, and 20%). The OD values and corresponding cell viability percentages showed a concentration-dependent decline in viability across all groups, with the most pronounced reduction observed in the Illusion^®^ Aligners group (Graph 1). Representative morphology (Figures 3–5) shows largely preserved fibroblast architecture despite differences observed in quantitative viability.

## Discussion

This investigation was designed to assess and contrast the harmful cellular effects of three widely used transparent orthodontic aligner brands – Invisalign^®^, Smile^®^ Aligners, and Illusion^®^ Aligners – on HGF cells by employing the MTT colorimetric test. Results showed notable differences in how each aligner affected cell survival, with Invisalign^®^ showing the least toxic response, Smile^®^ Aligners ranking second, and Illusion^®^ Aligners causing the greatest reduction in viable cells, especially as concentration increased (Table 1).

The superior biocompatibility of Invisalign^®^ may be attributed to its proprietary SmartTrack^®^ material, a medical-grade polyurethane-based thermoplastic designed specifically for orthodontic applications. The high cell viability (> 97%) across all concentrations suggests minimal leaching of any potentially toxic components. This is consistent with the findings of Eliades et al., who reported that Invisalign^®^ aligners exhibited low cytotoxic and estrogenic activity in vitro, with a chemical composition that remained stable over time [3].

Smile^®^ Aligners, composed primarily of PETG, also demonstrated acceptable cytocompatibility, though a slight decrease in cell viability was observed at higher concentrations. PETG is a commonly used thermoformable polymer in dental appliances and is generally considered safe; however, minor leaching under intraoral conditions has been reported in some studies [8]. The viability values observed in this study (>94%) are in agreement with Martina et al., who found that PETG-based aligners displayed non-cytotoxic effects on HGFs when tested under similar conditions [4].

In contrast, Illusion^®^ Aligners showed a statistically significant and concentration-dependent cytotoxic response. Cell viability dropped to 83% at 10% eluate concentration and further to 80% at 20% (Graph 1), classifying the material as slightly cytotoxic according to ISO 10993-5 guidelines [9]. The marked decrease in viability suggests that Illusion^®^ Aligners may contain a higher proportion of unreacted monomers or industrial-grade thermoplastics not optimized for intraoral use. This finding aligns with the results from Alhendi et al., who demonstrated that several regionally manufactured aligners exhibited significantly lower biocompatibility than established global brands [10].

The use of the MTT assay in this study provided a sensitive and reproducible measure of mitochondrial activity and overall cell viability. Although MTT is a reliable first-line screening tool, it does not distinguish between apoptotic and necrotic cell death, nor does it assess oxidative stress or inflammatory cytokine production. Therefore, while useful for comparative cytotoxicity assessments, the MTT assay should ideally be supplemented with complementary tests such as reactive oxygen species (ROS) assays or ELISA-based cytokine profiling in future studies.

Previous studies, including those by Premaraj et al. and Ozkan et al., have underscored the importance of simulating intraoral conditions during in vitro testing, noting that factors like salivary pH, enzymatic breakdown, and thermal cycling can influence the release of toxic substances from dental materials [5, 7]. While the current study attempted to mimic prolonged intraoral exposure by incubating samples in saline for 30 days, the static nature of this model still falls short of replicating the complex dynamics of the oral cavity.

The clinical implications of these findings are noteworthy. While all three tested aligner systems fell within acceptable biocompatibility limits, the observed variability highlights the need for clinicians to consider not just the mechanical performance and cost-effectiveness of aligner brands but also their biological safety. Moreover, long-term aligner use often involves refinements, mid-course corrections, or multiple appliance changes over extended treatment periods. This increases cumulative exposure to any potentially leached substances. In such cases, even slightly cytotoxic materials could produce cumulative adverse effects, particularly in sensitive patients.

Another consideration is the lack of transparency in material compositions among many local or unregulated aligner manufacturers. Without mandatory regulatory checks on biocompatibility and toxicity, there is an increased risk of clinical use of substandard materials, particularly as many emerging or low-cost aligner brands lack transparency regarding their material composition and do not provide publicly available biocompatibility data. In this context, routine in vitro screening of new aligner materials should become a regulatory requirement rather than an optional quality-control step.

## Conclusion

- Invisalign^®^ (Group A) consistently showed minimal cytotoxicity, with only 1–3% variability across concentrations. It is the most stable and biologically safe material in all inter-group comparisons. Hence, it may be considered for long-term treatments.
- Smile^®^ Aligners (Group B) showed moderate biocompatibility, with a clear, though not alarming, drop in viability at higher concentrations.
- Illusion^®^ Aligners (Group C) showed a clear dose-dependent cytotoxic profile and pose the greatest biological risk among the three systems, particularly at 10 and 20% concentrations, where viability approaches a critical threshold.

These results highlight the need for transparency in material composition and standardization in manufacturing protocols for all aligner systems to ensure patient safety.

The study was limited by short-term evaluation and a relatively small sample size, potentially affecting long-term extrapolation; however, cytotoxic effects are known to be most pronounced during early material exposure [14].

## Recommendations and future directions

- Further research should include additional biocompatibility tests such as ROS assays, cytokine profiling, and long-term exposure studies.
- In vivo models and clinical trials should be conducted to corroborate these in vitro findings.
- Regulatory authorities should consider implementing mandatory cytotoxicity testing for all orthodontic devices before market release, especially for local or emerging brands.
- Orthodontic clinicians are encouraged to critically evaluate not only treatment outcomes but also the biological performance of the materials they prescribe.

## Data Availability

The datasets generated and analyzed during the current study are available from the corresponding author, Dr. Parag Gangurde on reasonable request.
